# Fractal Anatomy of Human Organs: A Narrative Review of Structure, Function, and Clinical Perspectives

**DOI:** 10.1002/ca.70052

**Published:** 2025-11-25

**Authors:** Immacolata Belviso, Jacopo Junio Valerio Branca, Giulia Guarnieri, Annamaria Morelli, Alessandra Pacini, Daniele Della Posta, Domenico Ribatti, Ferdinando Paternostro

**Affiliations:** ^1^ Department of Psychology and Health Sciences Telematic University Pegaso Naples Italy; ^2^ Department of Experimental and Clinical Medicine University of Firenze Firenze Italy; ^3^ The Anatomical Network APS Rome Italy; ^4^ Department of Translational Biomedicine and Neuroscience University of Bari Medical School Bari Italy

**Keywords:** anatomy, clinical applications, computational anatomy, fractal dimension, fractal geometry, imaging biomarkers, organ systems

## Abstract

Fractal geometry describes complex, self‐similar patterns that repeat across spatial scales and is increasingly recognized as relevant in anatomical research. Indeed, the fractal organization is consistently observed in respiratory, cardiovascular, gastrointestinal, nervous, renal, hepatic, and dermatological systems. A comprehensive literature search was conducted on PubMed, Scopus, and Web of Science (1977 to March 2025) identifying peer‐reviewed original articles, reviews, and conference proceedings addressing the fractal organization of human organs at macrostructural or microstructural levels, with structural–functional relationships and/or clinical applications. Studies were excluded if they lacked direct translational relevance to humans, were not peer‐reviewed, or did not utilize explicit fractal methodology. Key findings highlight that bronchial tree fractal dimension (FD) correlates with airflow limitation in chronic obstructive pulmonary disease, while in the vascular system, retinal metrics reflect systemic microvascular health. Moreover, the fractal modeling of hepatic and renal hemodynamic models supports system‐level interpretation. In the nervous system, cortical gyrification and neuronal dendritic FD are associated with cognitive capacity and disease progression. Gastrointestinal mucosal FD decreases in inflammatory and neoplastic conditions. Advances in multiscale imaging (e.g., micro‐CT, MRI) and computational methods enable both in vivo and ex vivo assessment, although methodological heterogeneity remains a limiting factor. Overall, fractal analysis provides a quantitative and reproducible descriptor of anatomical complexity with demonstrated associations to functional performance and disease severity. Standardization of methodology, development of normative datasets, and validation in large prospective cohorts are essential for routine clinical practice.

## 1 Introduction

Fractal geometry, introduced by Benoit Mandelbrot (1924–2010), provides a mathematical framework for describing irregular, self‐similar structures recurring across spatial scales (Mandelbrot 1982). In anatomy, fractal organization is increasingly recognized as a fundamental design principle that optimizes space‐filling, branching, and exchange surfaces, and can be quantified using measures such as fractal dimension (FD) (Bassingthwaighte et al. 1994; Losa and Nonnenmacher 1996). Notably, FD plays a crucial role in assessing the efficacy of these branching systems in organs allowing predictions about their functional effectiveness and potential clinical implications.

Applications include the bronchial tree (Weibel 1991; Mishima et al. 1999; Bodduluri et al. 2018), vascular networks (Kamiya and Togawa 1980; Masters 2004; Grauslund et al. 2010; Farrah et al. 2020), cortical gyrification (Sowell et al. 2003), gastrointestinal villi (Iwabuchi et al. 2002; Grizzi et al. 2023), and cutaneous microvascular loops (Cutolo and Smith 2013). These structures demonstrate scale‐invariant behaviors promoting efficient transport while minimizing energetic cost. Alterations in physiological fractal patterns are associated with disease, such as reduced FD in chronic obstructive pulmonary disease (COPD), microvascular rarefaction in systemic sclerosis, dendritic simplification in neurodegeneration, or architectural distortion in inflammatory bowel disease (IBD) (Caserta et al. 1995; Iwabuchi et al. 2002; Sowell et al. 2003; Masters 2004; Lopes and Betrouni 2009; Cutolo and Smith 2013; Di Ieva et al. 2014; Grizzi et al. 2023).

Advances in imaging (micro‐CT [computed tomography], MRI [magnetic resonance imaging], confocal microscopy, retinal fundus photography) and computational methods enable multiscale assessment in vivo and ex vivo (Masters 2004; Lopes and Betrouni 2009; Debbaut et al. 2014). However, despite their potential, clinical integration remains limited due to methodological heterogeneity, lack of normative datasets, and insufficient validation in large cohorts. This review synthesizes the evidence on fractal organization across major human organ systems, underlining the structural–functional relationships and translational applications.

## 2 Methodological Approach

A narrative synthesis was conducted following recognized practices for non‐systematic reviews. A comprehensive search (1977 to March 2025) covered PubMed, Scopus, and Web of Science and the search terms included the combination of: fractal anatomy, fractal geometry, FD, fractal analysis, human organ, microvascular network, branching pattern, cortical folding, biomedical imaging, used in different combinations with and/or. No lower date limit was applied to ensure the inclusion of foundational works.

### 2.1 Eligibility Criteria

Studies were considered eligible if they examined fractal organization in human organs or tissues, provided quantitative analyses of fractal parameters (e.g., FD, multifractal spectrum), and explored structural–functional relationships and/or clinical applications. Works were excluded if they focused exclusively on non‐human models without explicit translational relevance, lacked a clear fractal methodology, or originated from non‐peer‐reviewed sources (such as preprints, theses, or nonacademic content). Historical experimental studies in animals were retained when they represented innovative and pioneering contributions that directly influenced subsequent research on human fractal physiology.

### 2.2 Study Selection and Data Extraction

Titles/abstracts were screened by two reviewers; full texts were assessed against inclusion criteria; extracted items included organ/system, imaging/modality, fractal method, principal findings, and clinical implications. Disagreements were resolved by consensus. This is a narrative review with a structured search and dual screening; no quantitative synthesis or meta‐analysis was undertaken.

While the narrative format allows a broad integrative perspective across diverse anatomical domains, it inherently carries a degree of selection bias, as inclusion and exclusion decisions may reflect interpretative judgment. To mitigate this limitation, the search was conducted across three major databases (PubMed, Scopus, and Web of Science), dual independent screening was performed, and only peer‐reviewed studies explicitly applying fractal methodology were retained. Nonetheless, we acknowledge that a fully systematic review with quantitative synthesis might yield additional insights.

### 2.3 Fractal Metrics

Beyond classical FD, many studies emphasize complementary measures such as lacunarity and the multifractal spectrum (Plotnick et al. 1993; Smith et al. 1996; Lopes and Betrouni 2009; Seuront 2009).

- Lacunarity quantifies spatial heterogeneity or “gappiness,” distinguishing textures with identical FD.
- The multifractal spectrum extends FD to scale‐dependent variations, especially in tumors and fibrotic tissues.

Both have been applied in neuroimaging, gastrointestinal pathology, oncology, and radiomics, enhancing reproducibility and diagnostic specificity.

### 2.4 Methods of Fractal Analysis in Anatomy

Fractal analysis quantifies geometric complexity and scale‐invariance by relating structural detail to observation scale. The principal parameter is the FD, a non‐integer measure of space‐filling capacity, branching complexity, and boundary irregularity (Mandelbrot 1982; Losa and Nonnenmacher 1996; Di Ieva et al. 2014).

*Box‐counting*: A grid of boxes of side ε is overlaid on a binary (or segmented) image; the number of occupied boxes *N*(*ε*) is recorded across scales and FD is obtained from the scaling law

$$N\left(\varepsilon \right)\propto \varepsilon^{-}ᴰ\;\text{with}\;D=-d\;\ln\;N\left(\varepsilon \right)/d\;\ln\;\varepsilon \;\left(\varepsilon \to 0\right)$$

Box‐counting is applicable to 2D/3D data but is sensitive to resolution, thresholding, and implementation details, which may limit reproducibility across software and sites (Mandelbrot 1982; Lopes and Betrouni 2009; Di Ieva et al. 2014; Losa and Nonnenmacher 1996).

To mitigate finite‐size effects, best practice includes the evaluation of local slopes across sliding windows in log–log plots and the objective selection of the scaling region (e.g., by maximizing linearity or *R* ^2^). Reporting both the range of scales analyzed and the criteria used for scaling‐region selection increases reproducibility and comparability across studies.

*Mass–radius (correlation) scaling*: The growth of mass/area within concentric circles/spheres as a function of radius is analyzed to assess space‐filling behavior; this suits radially organized structures such as vascular trees or optic‐disc‐centered retinal networks (Bassingthwaighte et al. 1994; Masters 2004; Lopes and Betrouni 2009).

*Perimeter–area relationship*: FD is inferred from the scaling between contour length and enclosed area, providing a contour‐based descriptor useful for histological boundaries; accuracy relies on precise segmentation and mitigation of pixelation bias (Smith et al. 1996; Lopes and Betrouni 2009).

*Minkowski–Bouligand (morphological dilation)*: Changes in area/volume as object boundaries are dilated by a structuring element of radius r yield FD estimates that capture 3D irregularity; the method is relatively robust to noise but computationally demanding and benefits from high‐quality imaging (Smith et al. 1996; Lopes and Betrouni 2009).

*Pipelines and multimodality*: Contemporary workflows integrate histology, confocal/multiphoton microscopy, micro‐CT, MRI/CT and retinal fundus imaging. Automated or semi‐automated pipelines—often coupled with machine learning—enable multiscale analysis but still suffer from heterogeneity in resolution, segmentation, and algorithmic parameters, underscoring the need for standardization (Masters 2004; Lopes and Betrouni 2009; Debbaut et al. 2014).

*Notation*: In formula, we write *D* for FD; in the text, we use FD. Within this section, *D* ≡ FD.

It is important to note that practical estimations obtained via box‐counting, correlation, perimeter–area, or Minkowski methods approximate different formal notions of FD (capacity, correlation, contour, morphological, respectively). These values may not coincide with the theoretical Hausdorff dimension, and slight discrepancies across studies often reflect such methodological distinctions rather than true biological variation.

For clarity and comparability, a summary table is provided (Table 1) outlining the main analytical methods, their formulas, required inputs, key advantages, limitations, typical anatomical applications, and seminal references.

**TABLE 1:** Summary of fractal analysis methods in anatomical studies.

Method	Formula/scaling law	Required input	Advantages	Limitations	Typical applications	Key references
Box‐counting	*N*(*ε*) ∝ *ε* ^−^ *ᴰ*	Binary/segmented 2D or 3D image	Simple, widely used	Sensitive to resolution, thresholding	Airways, cortical folds, histology	Mandelbrot (1982); Di Ieva et al. (2014)
Correlation (mass–radius)	*M*(*r*) ∝ *rᴰ*	Coordinates or images	Captures radial structures	Needs center definition	Retinal vessels, vascular trees	Bassingthwaighte et al. (1994); Masters (2004)
Perimeter–area	*P* ∝ *A* ^(*D*/2)^	Contours from histology	Useful for boundaries	Pixelation bias	Tumor margins, skin, cerebellum	Smith et al. (1996)
Minkowski dilation	*V*(*r*) ∝ *r* ^(*d*–*D*)^	Binary 2D/3D masks	Robust to noise	Computationally heavy	3D organs, liver sinusoids	Lopes and Betrouni (2009)
Multifractal	*τ*(*q*), *f*(*α*) spectra	Image intensities	Captures heterogeneity	Requires larger data	Tumors, fibrosis	Seuront (2009); Di Ieva et al. (2015)

## 3 Fractal Organization of Human Organs

### 3.1 Respiratory System

The human lung is one of the paradigmatic examples of fractal organization in anatomy. The bronchial tree follows an almost perfect dichotomous branching pattern that maximizes alveolar surface while minimizing energy dissipation and dead space. Morphometric studies beginning with Weibel (Weibel 1991) and later expanded with computational approaches (Mishima et al. 1999; Bodduluri et al. 2018) demonstrated that the FD of healthy bronchial trees typically ranges between ≈1.6 and 1.8, reflecting optimal balance between airway complexity and ventilatory efficiency.

Pathological changes are characterized by measurable deviations from this range. In COPD, terminal airspace simplification reduces FD, correlating with forced expiratory volume in 1 s (FEV_1_) decline and impaired gas exchange (Mishima et al. 1999). These findings support the potential role of airway FD as a biomarker for early detection, staging, and monitoring of respiratory disease progression.

### 3.2 Cardiovascular and Microvascular Systems

The cardiovascular system demonstrates fractal organization at multiple hierarchical levels: from large arterial branching, through arterioles and venules, down to the dense and apparently stochastic capillary networks. This arrangement minimizes hydraulic energy expenditure while ensuring uniform tissue perfusion (Masters 2004).

The retinal vasculature represents the most accessible human microvascular bed for noninvasive investigation. Retinal fundus photography combined with fractal analysis consistently shows that reduced vascular FD is associated with diabetes, hypertension, and increased cardiovascular risk (Masters 2004; Farrah et al. 2020). Large population studies confirm that retinal FD is associated with microvascular complications even before overt clinical symptoms, highlighting its translational potential as a systemic biomarker (Masters 2004; Cheung et al. 2009; Grauslund et al. 2010; Farrah et al. 2020).

In the renal system, fractal branching of interlobar and arcuate arteries continues into the glomerular loops, providing an efficient filtering interface consistent with flow‐adaptation principles. Clinical evidence also highlights eye–kidney microvascular correlations (Lim et al. 2009; Farrah et al. 2020).

In the liver, sinusoidal networks exhibit fractal organization that ensures efficient hepatocyte perfusion. Micro‐CT (Debbaut et al. 2014) and histology‐based fractal analyses (Gaudio et al. 2005) demonstrate detailed sinusoidal and vascular architecture and enable quantitative morphometry. These reconstructions are ex vivo and feasibility‐oriented; clinical translation will require standardized in vivo pipelines and reference datasets.

Fractal‐based analyses have demonstrated disease‐associated alterations in tissue architecture within histological models (Gaudio et al. 2005).

In the skin, capillaroscopic images reveal characteristic microangiopathic changes (e.g., giant capillaries, avascular areas) that correlate with disease severity (Cutolo and Smith 2013).

### 3.3 Gastrointestinal System

The gastrointestinal tract demonstrates fractal organization across multiple structural levels. The villus–crypt system of the small intestine and the glandular architecture of the colon both display scale‐invariant branching that maximizes absorptive and secretory surface area (Iwabuchi et al. 2002; Grizzi et al. 2023).

In IBD, alterations in mucosal architecture have been shown to translate into quantifiable changes in fractal parameters (FD, lacunarity), which show diagnostic implications and align with histopathological assessment (Grizzi et al. 2023); similar observations have been reported in methodological reviews of coeliac disease (Lopes and Betrouni 2009). Longitudinal studies suggest that recovery of mucosal FD accompanies effective therapy, supporting its potential as a quantitative tool for disease monitoring.

Adenomas, including serrated lesions, display characteristic increases in FD compared to normal mucosa (Iwabuchi et al. 2002); moreover, multifractal descriptors may add further discrimination across tumor types (Lopes and Betrouni 2009). These features provide diagnostic discrimination beyond conventional Euclidean morphometrics, supporting the value of fractal analysis for early detection and risk stratification of gastrointestinal neoplasia.

### 3.4 Nervous System

Fractal geometry permeates the nervous system, spanning from macroscopic folding of cortical surfaces to microscopic and subcellular structures.

- *Cortical gyrification*: MRI‐based studies have consistently demonstrated that cortical FD correlates with intelligence and cognitive capacity. Reductions in FD are observed with advancing age and are particularly marked in Alzheimer's disease, schizophrenia, and other neuropsychiatric conditions (Sowell et al. 2003; Di Ieva et al. 2014; Davidson et al. 2024).
- *Neuronal arborization*: Dendritic and axonal branching follow fractal patterns that optimize synaptic connectivity while minimizing wiring cost. Studies of neuronal dendrites and astrocytic processes reveal scale‐invariant patterns (Caserta et al. 1995; Di Ieva et al. 2014). Furthermore, at the subcellular level, organelle distribution and cytoskeletal organization also display fractal features, linking morphology to metabolic efficiency (Smith et al. 1996).
- *Cerebellum*: Contour‐scaling fractal analysis applied to the arbor vitae indicates that fractal descriptors capture the degree of cerebellar organization, with potential relevance to motor coordination (Maryenko and Stepanenko 2024).
- *Neurophysiology*: Fractal analysis of EEG signals provides functional correlates to structural organization. Both Alzheimer's disease and epilepsy are characterized by a reduced Higuchi FD and altered multifractal spectra, reflecting loss of network complexity (Affinito et al. 1997). These electrophysiological fractal measures complement structural MRI, enabling dynamic monitoring of neural integrity.

### 3.5 Clinical and Translational Perspectives

The clinical significance of fractal analysis lies in its ability to detect structural and functional alterations that often precede overt morphological damage detectable with conventional techniques. By quantifying complexity, space‐filling capacity, and branching efficiency, fractal metrics provide a unifying descriptor of anatomical organization that can be consistently applied across different imaging modalities and organ systems.

#### 3.5.1 Pulmonology

In respiratory medicine, the FD of the bronchial tree derived from high‐resolution CT imaging shows strong correlations with functional indices such as FEV_1_, total lung capacity, and diffusing capacity for carbon monoxide. In COPD, reductions in FD reflect the combined effects of alveolar destruction, small‐airway narrowing, and simplification of branching complexity (Weibel 1991; Mishima et al. 1999). Similar patterns are observed in idiopathic pulmonary fibrosis, where progressive declines in FD parallel both histopathological fibrosis and functional deterioration. Importantly, early reductions in airway FD can be detected before severe morphological destruction occurs, suggesting its potential role as a biomarker for early diagnosis, phenotyping, and longitudinal monitoring in both COPD and interstitial lung disease.

#### 3.5.2 Cardiology and Vascular Medicine

Fractal descriptors of the vasculature provide novel quantitative markers of systemic microvascular health (Masters 2004; Cheung et al. 2009). Retinal vascular FD has been extensively investigated as a noninvasive surrogate for microcirculatory integrity, with consistent associations with diabetes, hypertension, and increased cardiovascular risk (Masters 2004; Cheung et al. 2009; Grauslund et al. 2010; Farrah et al. 2020). Reductions in retinal FD have also been linked to stroke (Cheung et al. 2010) and chronic kidney disease, supporting its role as an integrative systemic biomarker (Cavallari et al. 2011; Farrah et al. 2020). In hepatology, fractal analysis of hepatic sinusoids demonstrates that decreasing FD accompanies progressive microvascular remodeling (Debbaut et al. 2014); these observations are consistent with classical flow‐adaptation principles (Kamiya and Togawa 1980). These observations highlight the potential for vascular FD to provide a global, noninvasive indicator of systemic health, with applications in risk stratification and early intervention.

#### 3.5.3 Neurology and Neuropsychiatry

The nervous system has been one of the most extensively investigated domains for fractal analysis. At the macroscopic level, cortical gyrification FD measured by MRI correlates with intelligence and cognitive reserve, whereas reductions are consistently observed in Alzheimer's disease, schizophrenia, and other neuropsychiatric disorders (Sowell et al. 2003; Di Ieva et al. 2014; Davidson et al. 2024). At the microscopic scale, dendritic arborization and astrocytic processes display fractal organization essential for synaptic connectivity; reductions in dendritic FD mirror synaptic loss in neurodegeneration (Caserta et al. 1995; Di Ieva et al. 2014). From a functional perspective, EEG‐derived fractal metrics, such as Higuchi FD and multifractal descriptors, capture alterations in neural signal complexity in conditions including epilepsy and dementia (Affinito et al. 1997). These electrophysiological measures complement structural imaging and may enable noninvasive monitoring of disease progression and therapeutic response. This evidence supports the use of fractal analysis as a bridge linking neural architecture and functional output.

#### 3.5.4 Gastroenterology and Oncology

In the gastrointestinal system, fractal analysis of mucosal architecture has demonstrated both diagnostic and prognostic value. Inflammatory conditions such as coeliac disease and IBD are characterized by measurable reductions in mucosal FD, reflecting villus blunting and crypt distortion (Grizzi et al. 2023). These changes are potentially reversible with successful therapy, positioning FD as a candidate biomarker for treatment response. In oncological applications, fractal and multifractal analysis of endoscopic and histopathological images improves discrimination between benign and malignant lesions. In the colorectum, FD discriminates adenoma subtypes including villous forms (Iwabuchi et al. 2002). More broadly, fractal descriptors applied to radiological and histological imaging across tumor types (breast, lung, brain, and gastrointestinal cancers) demonstrate higher diagnostic accuracy, particularly when combined with textural and radiomic analysis (Baish and Jain 1998; Li et al. 2007; Di Ieva et al. 2015). Collectively, these findings support a central role for fractal analysis in computational pathology and precision oncology.

#### 3.5.5 Dermatology and Rheumatology

In dermatology and connective tissue disorders, fractal metrics derived from capillaroscopy and skin (histo)dermoscopy provide sensitive markers of disease activity. In systemic sclerosis, nailfold capillaroscopy reveals characteristic microangiopathy that correlates with clinical severity and disease progression (Cutolo and Smith 2013). In dermatology, fractal metrics have been applied to skin morphology: in psoriasis, the FD of the dermo‐epidermal junction is higher in plaques than in guttate lesions and uninvolved skin (Uhoda et al. 2005). These approaches are reproducible, relatively inexpensive, and suitable for serial monitoring, supporting their potential for clinical practice.

### 3.6 Cross‐System Integration

Perhaps the most significant translational potential of fractal analysis lies in its universality. The same mathematical framework can be applied across different modalities, including lung CT, retinal fundus photographs, brain MRI, liver micro‐CT, gastrointestinal histology, and skin capillaroscopy. This cross‐modality adaptability allows for a systems‐level perspective, where alterations in one organ system may mirror systemic pathophysiology. For example, retinal vascular FD may serve as a proxy for systemic microvascular health, while cortical and dendritic FD reductions may align with neurocognitive decline.

Moreover, the integration of fractal descriptors with functional measures, such as spirometry in pulmonology, EEG in neurology, elastography in hepatology, or flowmetry in vascular medicine, may offer the potential to generate composite biomarkers that are more powerful than either modality alone. Combined with advances in artificial intelligence and radiomics, automated pipelines could deliver rapid, reproducible, and clinically actionable fractal analysis across multiple medical specialties.

## 4 Discussion

We use the term “morphological transduction” in a conceptual, hypothesis‐generating sense: variations in anatomical form are posited to mediate, constrain, and reflect the transmission of physiological flows and forces across multiscale networks. Under this view, fractal descriptors—such as FD, lacunarity, and multifractal spectra—serve as quantitative indices of the efficiency and robustness of structure–function coupling. This interpretation is consistent with work linking fractal vascular architecture, transport efficiency, and angiogenesis (Baish and Jain 1998) and with translational applications that relate fractal organization to functional performance across organ systems (Di Ieva et al. 2015). We adopt the term solely as an interpretive framework to organize findings; it is not a standardized construct and does not replace disease‐specific mechanistic models.

Fractal analysis has emerged as a unifying framework to quantify anatomical complexity across organ systems, offering both a morphometric descriptor and a potential functional biomarker. The fact that fractal organization recurs across diverse tissues and organs, supports the hypothesis that it represents a fundamental principle of biological design, optimizing the trade‐off between maximal surface area, minimal transport distance, and metabolic efficiency (Mandelbrot 1982; Bassingthwaighte et al. 1994; Losa and Nonnenmacher 1996). The presence of consistent scaling behavior across multiple orders of magnitude, from macroscopic organ geometry to microscopic cellular arborization, suggests that fractal organization may arise from generic developmental constraints, such as morphogen diffusion, mechanical tension fields, and genetically regulated branching programs (Bassingthwaighte et al. 1994; Caserta et al. 1995).

However, it is important to emphasize that most biological structures are only approximately fractal and exhibit scale‐limited self‐similarity. True mathematical fractality, defined by infinite scaling, is rarely observed in vivo. Instead, biological tissues often display fractal‐like behavior across restricted spatial ranges constrained by developmental, genetic, and mechanical factors. Recognizing these deviations is essential, as they may modulate both functional interpretation and clinical applicability of fractal descriptors.

A key translational aspect is the sensitivity of fractal metrics to subtle pathological changes. In the lung, reductions in FD precede overt architectural destruction, implying that fractal analysis might enable earlier detection of small‐airway and alveolar injury in COPD or fibrotic lung disease (Mishima et al. 1999). Similarly, in the retina, FD can decline before clinically detectable microaneurysms or hemorrhages appear, making it a candidate screening tool for microangiopathy in high‐risk populations such as diabetics and hypertensives (Masters 2004; Farrah et al. 2020). In the brain, gyrification complexity is related to neurodevelopmental processes, and its reduction in neurodegenerative disorders aligns with the loss of synaptic and dendritic complexity seen at the histological level (Caserta et al. 1995; Sowell et al. 2003; Di Ieva et al. 2014; Davidson et al. 2024). These multiscale correlations support the notion that FD reflects not just static morphology but also the functional capacity of an organ. Indeed, across multiple systems described in this review, variations in FD occur in conditions where the efficiency of physiological force transmission is impaired, from altered airflow propagation in COPD (Mishima et al. 1999) to disrupted portal flow in hepatic fibrosis (Debbaut et al. 2014), reduced filtration gradients in renal disease (Farrah et al. 2020), compromised synaptic signaling in neurodegeneration (Di Ieva et al. 2014; Davidson et al. 2024), and diminished microvascular perfusion in systemic sclerosis (Cutolo and Smith 2013). This supports the view that fractal metrics can be interpreted not only as static morphological descriptors but also as quantitative correlates of morphological transduction, here defined as the process by which variations in anatomical form mediate and reflect the transmission of physiological forces across interconnected structural modules (Baish and Jain 1998; Di Ieva et al. 2015).

From a methodological standpoint, the diversity of computational approaches, such as box‐counting (Mandelbrot 1982; Lopes and Betrouni 2009; Di Ieva et al. 2014), mass–radius (Bassingthwaighte et al. 1994; Masters 2004), perimeter–area, morphological dilation, and advanced multimodal integrations (Smith et al. 1996; Lopes and Betrouni 2009), presents both opportunities and challenges. On the one hand, the ability to tailor the analytical method to the geometry and scale of the target structure increases applicability. On the other hand, the lack of cross‐platform standardization in segmentation thresholds, resolution requirements, and algorithm implementation can lead to non‐comparable FD values between studies.

Indeed, the apparent discrepancies in reported FD values across studies often stem from methodological heterogeneity rather than genuine biological differences. For example, box‐counting and perimeter‐area methods capture distinct aspects of spatial complexity (capacity vs. contour), while Minkowski–Bouligand dilation emphasizes volumetric irregularity. Variations in pre‐processing steps (e.g., binarization, skeletonization, thresholding) further affect outcomes. As such, cross‐study comparability is limited unless acquisition parameters and computational pipelines are harmonized. Readers should interpret inter‐study differences with caution, considering the specific analytical approach employed.

To enhance reproducibility and cross‐study comparability, we recommend specifying minimal methodological prerequisites, including voxel size or pixel resolution, segmentation and binarization criteria, use of skeletonization, and any morphological operations (e.g., erosion, dilation) applied prior to analysis. The adoption and open sharing of standardized, open‐source pipelines would facilitate multicentric validation and benchmarking.

For instance, all airway measurements are influenced by acquisition volume, display field of view, and CT spatial resolution. Thus, increasing efforts are underway to standardize CT acquisition protocols to reduce the impact of these variables (Horsfield 1990; Bodduluri et al. 2018).

Another important theme is the potential of fractal analysis to inform mechanistic models of disease. In hepatic fibrosis, progressive loss of sinusoidal complexity (Debbaut et al. 2014) may not merely reflect architectural collapse but also the redistribution of shear stress and altered portal–central blood flow, linking morphometric parameters directly to hemodynamic consequences. In renal disease, flow‐adaptation principles align with evidence of decreased microvascular complexity in hepatic models, which may by analogy parallel maladaptive vascular remodeling and nephron dropout; this framework was modeled by Kamiya and Togawa (1980) and demonstrated in hepatic microvasculature (Gaudio et al. 2005; Debbaut et al. 2014). In oncology, multifractal analysis has revealed scale‐dependent changes in tumor architecture (Baish and Jain 1998; Li et al. 2007), suggesting that malignant transformation involves both local irregularity and altered global organization.

The integration of structural fractal metrics with functional measures (e.g., spirometry, EEG complexity, retinal flowmetry) may produce composite biomarkers that outperform either alone. In neurology, combining cortical FD from MRI with EEG Higuchi‐FD (Affinito et al. 1997) could yield a more complete picture of neural reserve and network integrity. In gastroenterology, coupling mucosal FD with permeability assays could help stratify IBD activity (Iwabuchi et al. 2002; Grizzi et al. 2023).

Although multiple studies demonstrate promising associations between FD and disease severity across organ systems, the majority are cross‐sectional, exploratory, and based on modest sample sizes. Longitudinal validation, assessment of intra‐ and inter‐operator reproducibility, and establishment of normative reference ranges remain largely incomplete. Therefore, the translational readiness of fractal analysis should be viewed as preliminary; current evidence supports its potential as a research and hypothesis‐generating tool rather than an established clinical biomarker.

Despite these advances, several gaps remain. Normative datasets stratified by age, sex, ethnicity, and physiological variables are largely absent, yet essential for interpreting individual patient values. Longitudinal studies to determine whether therapeutic interventions can restore or stabilize FD, such as anti‐fibrotic agents in liver disease or neuroprotective drugs in dementia, are rare (Debbaut et al. 2014; Davidson et al. 2024). Furthermore, most current analyses are cross‐sectional; dynamic fractal analysis of real‐time imaging sequences could open new avenues for functional assessment (Baish and Jain 1998; Di Ieva et al. 2015).

### 4.1 Methodological Limitations and Future Directions

This review is narrative in nature, which, despite a structured search and dual screening, introduces potential selection bias. Heterogeneity in fractal metrics, imaging modalities, and computational pipelines limits direct comparability of reported FD values. Most available studies are cross‐sectional and exploratory, with scarce longitudinal validation or standardization. Furthermore, fractal organization in biology is typically approximate and scale‐limited rather than mathematically ideal. These constraints underscore the need for consensus guidelines, multicentric reproducibility studies, and harmonized open‐source analysis pipelines to enable robust clinical translation.

## 5 Concluding Remarks

Fractal analysis offers a universal, quantitative framework for linking anatomical form and function across scales. Deviations from physiological fractal patterns are detectable in multiple organ systems and often precede gross structural damage, supporting their role as candidate early biomarkers. With methodological standardization, normative datasets, and integration into multimodal diagnostics, potentially enhanced by AI and radiomics, fractal metrics could become powerful tools for personalized medicine, guiding diagnosis, monitoring, and outcome prediction across different disorders.

Future consensus guidelines and standardized protocols will be essential to fully realize the translational potential of fractal anatomy.

## Funding

The authors have nothing to report.

## Ethics Statement

As a narrative review, it involves no human/animal subjects, interventions, or identifiable data.

## Consent

The authors have nothing to report.

## Data Availability

Data sharing not applicable to this article as no datasets were generated or analysed during the current study.
